# Development of Rapid Isothermal Detection Methods Real-Time Fluorescence and Lateral Flow Reverse Transcription Recombinase-Aided Amplification Assay for Bovine Coronavirus

**DOI:** 10.1155/2024/7108960

**Published:** 2024-02-10

**Authors:** Qingqing Li, Yan Pan, Cuilan Wu, Chunxia Ma, Jun Li, Changting Li, Huili Bai, Yu Gong, Jing Liu, Li Tao, Yangyan Yin, Ling Teng, Shuhong Zhong, Meiyi Lan, Shuai Hu, Xiongbiao Xuan, Tianchao Wei, Hao Peng

**Affiliations:** ^1^College of Animal Science and Technology, Guangxi University, Nanning 530004, China; ^2^Guangxi Key Laboratory of Veterinary Biotechnology, Guangxi Veterinary Research Institute, Nanning 530001, China; ^3^College of Animal Science and Technology, Guangxi Vocational University of Agriculture, Nanning 530007, China; ^4^Key Laboratory of China (Guangxi)-ASEAN Cross-Border Animal Disease Prevention and Control, Ministry of Agriculture and Rural Affairs of China, Nanning, China; ^5^Guizhou Animal Husbandry and Veterinary Research Institute, Guiyang 550005, China

## Abstract

Bovine coronavirus (BCoV) is a notable pathogen affecting newly born calves and adult cattle, increasing mortality rates among calves and reducing productivity in meat and dairy industries, thereby causing substantial economic losses. Current primary laboratory methods for detecting BCoV include RT-PCR assay, real-time RT-PCR assay, and ELISA. However, these methods are time-consuming, require specialized technicians, and necessitate a laboratory environment. Consequently, there is an urgent need for a rapid, sensitive, and easy to use diagnostic method to detect BCoV. This study introduces two innovative protocols: the real-time fluorescent reverse transcription recombinase-aided amplification (RT-RAA) and the test strip RT-RAA (RT-RAA-LFD). Our results indicate that real-time RT-RAA can complete the reaction in 20 min at 39°C, while RT-RAA-LFD can achieve detection in just 17.5 min at 35°C. These new approaches offer higher specificity, with no cross-reactivity to other viruses, and significantly enhanced sensitivity compared to existing methods (1.46 × 10^1^ and 1.46 × 10^2^ copies/*μ*L, respectively). We evaluated the performance of our methods using 242 clinical samples, and compared with RT-PCR and RT-qPCR. Both real-time RT-RAA and RT-qPCR yielded similar detection rates, the detection rate of RT-RAA-LFD was better than RT-PCR. The RT-RAA methods developed in this study effectively overcome the limitations associated with both RT-PCR and RT-qPCR by offering advantages including a single, low reaction temperature that allows for room temperature operation. Both methods boast shorter reaction times, simpler and more portable instrumentation, as well as reduced technical and environmental demands. Generally, both RT-RAA methods established in this study offer new avenues for the rapid detection of BCoV, contributing significantly to the monitoring, prevention, and control of the disease in global bovine industry.

## 1. Introduction

Bovine coronavirus (BCoV), a significant pathogen within livestock industry, is known to cause both digestive and respiratory diseases among cattle [1, 2]. Classified as a single-stranded, positive-sense RNA virus, BCoV falls under a subtype of the *β-coronavirus* genus within the *Coronaviridae* family. The virus has an encapsulated structure and appears as a rounded, spine-like shape under electron microscopy. Remarkably, BCoV has a genome size ranging between 27 and 32 kb, making it the largest known RNA virus to date [3, 4]. BCoV is implicated in a range of bovine diseases, including calf diarrhea (CD) in young calves aged 1–3 weeks, winter dysentery (WD) in adult cattle, and various respiratory illnesses. These conditions lead to diminished productivity in adult cattle, reduced milk production in dairy cows, and weight loss in cows raised for beef [5, 6]. BCoV is highly contagious, spreading through both respiratory, and digestive tracts. It is transmitted primary via the fecal-oral route, aerosols, and by asymptomatic carriers within herds. The virus is detectable in various samples, including nasal swab, lung, intestinal, and fecal specimens. BCoV strains are categorized into respiratory and intestinal types based on their clinical manifestations. Coinfects with other pathogens, such as bovine enteroviruses (BEV), BVDV, bovine rotavirus (BRV), *E. coli*, *Campylobacter*, *Salmonella*, *Clostridium perfringens*, *Cryptosporidium*, *Coccidioides*, *Ascaris*, and various other bovine diarrhea-causing, bovine respiratory syncytial virus (BRSV), bovine parainfluenza virus type 3 (BPIV3), bovine epidemic fever virus (BEFV), bovine infectious rhinotracheitis virus (IBRV), *Mycoplasma bovis*, *Pasteurella multocida*, *Mannheimia haemolytica*, and bovine herpesvirus type 1 (BHV-1), significantly elevate the risk of mortality. These coinfections can intensify disease severity, potentially leading to morbidity rates as high as 100% and mortality rates up to 50% [7–13].

The genomes of the *Coronaviridae* family are susceptible to mutations and recombination, which lead to genetic evolution and the emergence of new strains. These genetic changes can lead to altered host ranges, cross-species transmission, and variations in viral tissue tropism [4, 14–16]. Notably, members of the *β-coronavirus* genus, such as the BCoV, have demonstrated the ability to overcome interspecies barriers, facilitating cross-species transmission [17–20]. A striking example of this interspecies transmission was observed in 1988 when a coronavirus closely related to BCoV was isolated from the diarrheal feces of a pediatric patient in Germany, subsequently named Human Enteric Coronavirus (HEC 4408) [21]. Further evidence of this interspecies capability was seen in 1997, when Majhdi et al. [22] isolated the elk coronavirus (ECV) which showed a high degree of homology (99%) with BCoV in the nucleoprotein gene sequences. In 2003, Saif and Jung's [2] isolation of a coronavirus from a giraffe revealed a bovine-like COVID-19 capable of transmission from wild ruminants to cattle, though it exhibited some genetic differences from BCoV [23]. BCoV has a remarkably broad host range, encompassing goats, cows, horses, elk, giraffes, camels, dogs, cats, and humans [17, 18, 20]. To date, six human-infecting coronaviruses have been identified: HCoV-229E, HCoV-NL63, MERS-CoV, SARS-CoV, HCoV-OC43, and HCoV-HKU1. Four of these, namely MERS-CoV, SARS-CoV, HCoV-OC43, and HCoV-HKU1, belong to the *β-coronavirus* family [24, 25]. Intriguingly, BCoV and the common human cold coronavirus (HCoV-OC43) share the same cell-binding receptor, N-acetyl-9-O-acetylneuraminic acid. Evolutionary analyses have revealed a 96% homology between these two strains, suggesting a possible rodent origin for HCoV-OC43, which might have spread to humans via cattle [26–28]. The significant risk BCoV poses to the global cattle industry and its potential threat to public health cannot be overstated. As such, early detection and monitoring of BCoV in cattle farms are imperative to prevent outbreaks and limit the virus's spread. Therefore, developing of efficient detection methods for BCoV is of great importance.

**Figure 1:**
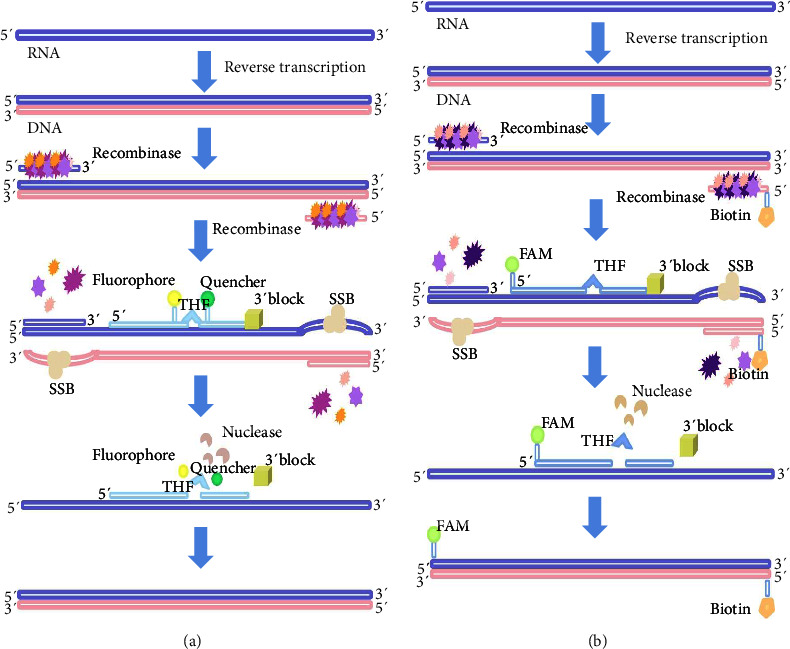
Schematic diagram of the RT-RAA assays for the detection of BCoV N gene. (a) The flowchart outlines the process for the real-time RT-RAA assay. (b) The RT-RAA-LFD test procedure. The fluorescent probe requires the insertion of an antigen marker, FAM, at the 5′ end, and a C3-spacer block at the 3′ end. An antigen marker, biotin, is also inserted at the 5′ end of the downstream primer. The amplified product is labeled with both FAM and biotin. SSB, single-stranded binding protein; FAM-dT, thymidine nucleotide carrying fluorescein; THF, tetrahydrofuran spacer; BHQ1-dT, thymidine nucleotide carrying black-hole Quencher-1; C3 spacer, polymerase extension blocking group; FAM, antigenic marker carboxyfluorescein.

The recently developed reverse transcription recombinase-aided amplification (RT-RAA) assay, known for its high sensitivity and specificity, has been successfully employed in detecting various pathogens. In the RT-RAA assay, RNA is first reverse-transcribed into DNA by a reverse-transcriptase enzyme. Subsequently, recombinase enzymes typically sourced from bacteria or fungi, and primers are tightly bound to form a recombinase–primer complex at room temperature. The primer firmly binds to create a recombinase–primer complex, which invades the double-stranded DNA nucleic acid template and initiates the unwinding of the double helix. Concurrently, single-stranded binding proteins attach to the now-exposed single-stranded DNA, maintaining the DNA in the open-stranded state. When the primer locates its perfectly matching complementary sequence on the template, the recombinase–primer complex disassembles, allowing the synthesis of a new double-stranded DNA segment facilitated by DNA polymerase. This process results in the amplification of the target gene (Figures 1(a) and 1(b)). The RAA assay can be integrated with various technologies such as agarose gel electrophoresis, fluorescent probes, lateral flow chromatography, CRISPR-Cas systems, and microarrays, as noted in [29–31]. However, there are no any reports on the detection of BCoV by real-time RT-RAA assay and RT-RAA-LFD assay. Therefore, we established real-time RT-RAA assay and RT-RAA-LFD assay for BCoV detection.

In this study, we developed two methods for detecting BCoV: the real-time reverse transcription recombinase aid amplification (RT-RAA) method and the RT-RAA-lateral flow dipstick (LFD) method. These methods utilize primers and probes tailored to the conserved sequences of the BCoV *N* protein gene. We evaluated and compared the specificity, sensitivity, and reproducibility of our RT-RAA methods against the established reverse transcription polymerase chain reaction (RT-PCR) and quantitative RT-PCR (RT-qPCR), which employs SYBR Green I. Testing on 242 bovine fecal, nasal swab, and tissue samples, we determined that both RT-RAA methods could be completed in under 20 min. Furthermore, the outcomes are easily discernible, either through fluorescence in real-time RT-RAA or via test strips in the RT-RAA-LFD approach.

**Figure 2:**
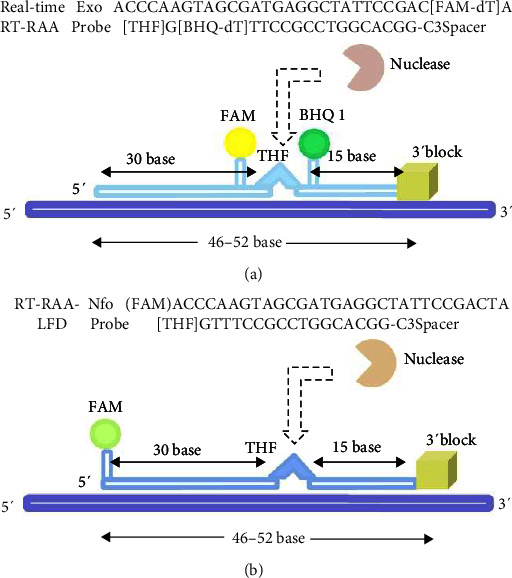
Principle of the probe design for the RT-RAA assay. (a) Principle of the probe design for real-time RT-RAA assay. Nuclease, nucleic acid exonuclease. (b) Principle of the probe design for RT-RAA-LFD assay. Nuclease, nucleic acid nfonuclease.

The primers and fluorescent probes used in the two RT-RAA assays are specifically designed for their unique roles. RT-RAA primers typically range from 28 to 35 bp, as primers exceeding 45 nucleotides may produce secondary structures and potential priming artifacts. The probes, approximately 46–52 nucleotides, incorporate tetrahydrofuran (THF), a nucleic acid exonuclease recognition site, are strategically positioned at a distance of at least 35 bases from the 5′ end and at least 15 bases from the 3′ end. The fluorescent probe (exo) of real-time RT-RAA needs to be labeled with a fluorescent group and a quenching group upstream and downstream of the THF site, respectively, with a blocker C3-spacer designed at the 3′ end. During the reaction, the nucleic acid exonuclease III cleaves tetrahydrofuran in the fluorescent probe, which releases fluorescent groups and quenching groups to generate fluorescent signals (Figure 2(a)). For the RT-RAA-LFD assay, the fluorescent probe (nfo) is modified by adding a fluorescent antigen marker FAM at the 5′ end and design a blocker C3-spacer at the 3′ end, as well as the insertion of an antigen marker biotin at the 5′ end of the downstream primer, resulting in an amplification product being labeled with both FAM and biotin (Figure 2(b)).

## 2. Materials and Methods

### 2.1. Samples and Viral Strains from Animals

To assess the specificity of deployment methods, a number of commonly implicated pathogens in bovine intestinal and respiratory disorders were chosen for evaluation. In the sample test results, BCoV was observed to be most frequently coinfected with over two viral species, including BVDV, BEV, BRV, BEFV, and BPIV 3, etc. Notably, among these, BEV and bovine @viral diarrhea virus (BVDV) emerged as two of the most prevalent coinfecting viruses. Consequently, considering both the existing literature [7–13] and the empirical findings from the study, these specific viruses were selected as controls. The BCoV positive reference strain, designated BCoV-GX-NN190313 (GenBank accession number MK757492.1), along with strains of BVDV, BEV, BRV, BEFV, and BPIV3, were isolated and subsequently preserved in our laboratory.

A total of 242 samples, which included 162 samples of diarrheal feces, 50 nasal swab samples, and 30 tissue samples, were collected over a period from June 2021 to December 2023. The unique specialization of our laboratory predominantly involves the analysis of fecal samples from farms. Given the prevalence of diarrheal diseases affecting cattle production, fecal samples represent the most commonly submitted specimen for testing due to their higher incidence rate of such conditions. In contrast, nasal swabs are less frequently collected, and lung tissue samples are typically procured postmortem, which significantly reduces their availability for study. These samples were sourced from local cattle farms in the following cities: Nanning, Chongzuo, Guigang, and Guilin, located in the Guangxi Zhuang Autonomous Region. Samples were transported at 4°C and stored at −80°C.

### 2.2. Sample Processing and RNA Extraction

Stool samples were mixed with PBS buffer at a ratio of either 1 : 5. Tissue samples were clipped, ground, combined with PBS, and thoroughly mixed before being dispensed into EP tubes. All samples were vortexed for complete mixing and stored at −80°C. The samples were then centrifuged at 8,000 rpm at 4°C for 10 min. Supernatant was extracted and prepared according to the manufacturer's instructions for the viral DNA/RNA extraction kit. All prepared RNA and DNA samples were stored at −80°C.

### 2.3. Generation of RNA Molecular Standard

The BCoV N gene (1440 bp) was amplified based on the BCoV-GX-NN190313 strain (GenBank accession number MK757492.1), and the amplified products were ligated into the pGEM-T Easy vector and transformed into DH5*α* cells (Sangong Bioengineering Co., Ltd., Shanghai, China). The products were then sent for sequencing analysis to confirm successful ligation to the vector. The RNA was transcribed in vitro using the SP6/T7 Transcription Kit and quantified with the Quant-iTTM RiboGreen RNA Assay Kit according to the manufacturer's instructions.

The copy number of the plasmid was calculated as follows: copy number (copies/*μ*L) = 6.02 × 10^23^ × concentration (ng/*μ*L) × 10^−9^/number of bases × 660. The standard plasmid copy number was determined to be 1.46 × 10^10^ copies/*μ*L.

### 2.4. Design of the RT-RAA Primers and Probes

To discern the most highly conserved region of the *N* gene of BCoV, we employed the DNAstar MegAlign software to compare the full-length *N* gene sequences available in the GenBank database. The *n*-terminal portion of the *N* gene was identified as the most highly conserved region and was thus utilized as the target for the formulation of primers. Three pairs of upstream and downstream primers, as well as one fluorescent probe, were designed using Oligo 7 and Primer Premier 5 software (Table 1). Primers and probes for both methods were synthesized by Shanghai Shengong Bioengineering Co., Ltd.

### 2.5. Screening and Optimization of Real-Time RT-RAA Reaction Conditions

**Figure 3:**
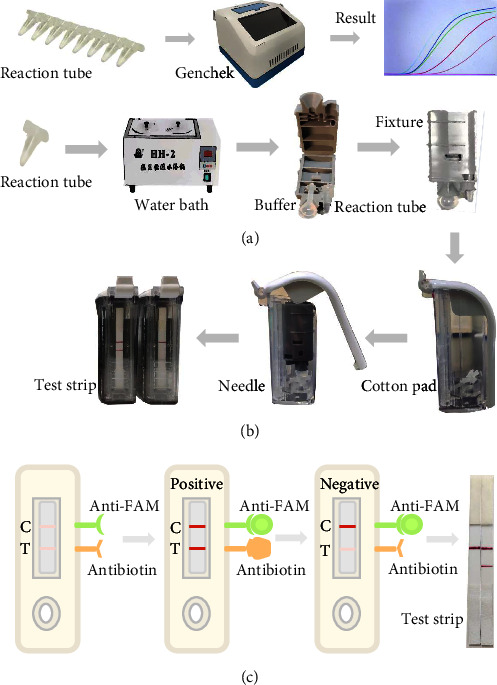
Procedure for the RT-RAA assays. (a) Procedure for real-time RT-RAA assay. (b) Procedure for RT-RAA-LFD assay. (c) The leading end of the strip carries a gold nanoparticle, with a FAM antibody on the C-line and a biotin antibody on the T line. C, quality control line; T, test line; Anti-FAM, anti-FAM antigen marker; Anti-Biotin, anti-biotin antigen marker.

The real-time RT-RAA reaction kit (Hangzhou Zhongmei Biological Co., Ltd., China) was used as per the manufacturer's instructions. The reaction system comprised 25 *μ*L of A Buffer, 15.3 *μ*L of ddH_2_O, 2.2 *μ*L each of upstream and downstream primers (10 *μ*M), 0.8 *μ*L of fluorescent probe (10 *μ*M), and 2.0 *μ*L of template. Magnesium acetate (2.5 *μ*L) was added dropwise to the cap of the reaction tube. All tubes were gently mixed by inverting 8–12 times and then centrifuged for 30 s. The tubes were placed in a Genchek real-time fluorescence thermostatic detector, and the reaction was conducted for 20 min at 39°C on the FAM channel. Fluorescence signals were collected every 30 s (Figure 3(a)).

For optimization, a standard RNA template with a concentration of 1.46 × 10^5^ copies/*μ*L was used in the test, with ddH_2_O serving as the negative control. We assessed nine primer pairs by combining three upstream and three downstream primers. The lengths of the respective primer pairs are as follows: F1/R1 : 175 bp, F1/R2 : 202 bp, F1/R3 : 247 bp, F2/R1 : 166 bp, F2/R2 : 193 bp, F2/R3 : 238 bp, F3/R1 : 144 bp, F3/R2 : 171 bp, and F3/R3 : 216 bp. The optimal primer pairs were selected based on their early peak times and high peak values, indicating efficient amplification. Various temperatures (33, 35, 37, 39, and 41°C), primer volumes (1.6, 1.8, 2.0, 2.2, and 2.4 *μ*L), and probe volumes (0.2, 0.4, 0.6, 0.8, and 1.0 *μ*L) were tested in this experiment.

### 2.6. Determination of RT-RAA-LFD Reaction Conditions

According to the RT-RAA-LFD kit manual (Hangzhou Zhongtai Biological Co., Ltd., China), a 50 *μ*L reaction system was prepared, as shown in Table 2. The system included 25 *μ*L of A Buffer, 15.9 *μ*L of ddH_2_O, 2.0 *μ*L each of upstream and downstream primers (2 *μ*M), and 0.6 *μ*L of fluorescent probe (2 *μ*M). All reagents were mixed, and 2.0 *μ*L of the template was added to the reaction tube, along with 2.5 *μ*L of acetic acid to the cap. The tube was inverted 8–12 times and briefly centrifuged for 30 s at room temperature. The sample was then incubated at 35°C for 12.5 min. After incubation, the reaction product was transferred to a device equipped with test strips. Results were observed 5 min after applying firm pressure to the handle of the device (Figure 3(b)).

The device comprises a fixing mechanism for the reaction tank, a vial of buffer to dilute the reaction product, a blade to cut the reaction tank, a needle to puncture the buffer vial, a test strip, and a cotton pad. The EP tube, carrying the reaction product, was initially affixed to the fixing device, which was then placed inside an airtight container holding the test strip. Both the EP tube and the buffer vial were punctured by forcefully pressing the handle, enabling the reaction product and buffer to flow onto the test strip via the cotton pad. The LFD has gold nanoparticles with FAM antibody on the front control line (*C*) and biotin antibody on the test line (*T*). When the product is dropped onto the test strip, the FAM motif at the *C* line is bound to the FAM antibody, and the biotin antibody at the *T* line is bound to the biotin on the amplified product. Eventually, the captured product shows a red band on the *C* and *T* lines, and the uncaptured product shows a red band on the *C* line. The results were interpreted based on the appearance of the *T* line and *C* line. A result was considered positive when both *T* and *C* lines turned red; negative when only the *C* line was visible; and invalid if the *C* line did not appear (Figure 3(c)).

LFD assays were conducted using the primer pairs selected from the previous screening to optimize primer concentration, reaction temperature, and reaction time. The reaction template was consistent with the one used earlier. Primer and probe concentrations were tested at five different levels (2, 4, 6, 8, and 10 *μ*M), temperatures were set at six different points (33, 35, 37, 39, 41, and 43°C), and reaction times were assessed at seven intervals (5, 7.5, 10, 12.5, 15, 17.5,and 20 min).

### 2.7. Real-Time RT-RAA Sensitivity, Specificity, and Reproducibility Assays

To analyze the sensitivity of real-time RT-RAA, the prepared standard RNA concentration was diluted in ranging from 1.46 × 10^10^ to 1.46 × 10^0^ copies/*μ*L. Concentrations ranging from 1.46 × 10^7^ to 1.46 × 10^0^ copies/*μ*L were selected as templates and ddH_2_O as the negative control, the optimized system was employed to perform real-time RT-RAA experiments. The sensitivity of the three methods was evaluated by comparing the real-time RT-RAA results with those of RT-PCR and RT-qPCR.

For the specificity assessment of the real-time RT-RAA assay, the potential for cross-reactivity was evaluated by detecting RNA or DNA of BCoV, BVDV, BEV, BRV, BEFV, and BPIV3.

Reproducibility was assessed using standard RNA concentrations of 1.46 × 10^7^, 1.46 × 10^5^, and 1.46 × 10^3^ copies/*μ*L. Each concentration was repeated three times with ddH_2_O serving as the negative control, and the reproducibility of the results was evaluated.

### 2.8. RT-RAA-LFD Sensitivity, Specificity, and Reproducibility Assays

The prepared standard RNA concentrations ranging from 1.46 × 10^7^ to 1.46 × 10^0^ copies/*μ*L were selected as templates. With ddH_2_O as a negative control, RT-RAA-LFD assays were performed, and the results were compared with those of RT-PCR and RT-qPCR to assess the detection sensitivity. The specificity and reproducibility of the RT-RAA-LFD assay were subsequently evaluated using the approach, as described in Section 2.6.

### 2.9. BCoV RT-PCR and RT-qPCR Sensitivity Assays

Sensitivity tests for RT-PCR and RT-qPCR were reported in the previous article [32–33]. For the RT-PCR assay, the following upstream primer P1(5′-GAGCGTCCTTTGGAAA-TCGT-3′) and downstream primer P2 (5′-GCTTAGTTACTTGCTGTGGC-3′) were utilized to generate an amplified fragment of 730 bp in length. The total reaction volume employed was 25 *μ*L, comprising 12.5 *μ*L of 2x Es Taq MasterMix (Dye), 1 *μ*L each of upstream and downstream primers (10 *μ*M), 2 *μ*L of template, and 8 *μ*L of ddH_2_O. The PCR reaction conditions were as follows: predenaturation at 95°C for 3 min, denaturation at 95°C for 30 s, annealing at 56°C for 30 s, and extension at 72°C for 10 min for 32 cycles. Results were visualized by electrophoresis on a 1.5% agarose gel.

For the RT-qPCR assay, the following upstream primer P3 (5′-TCGTTCTGGTAATGG-CATCCT-3′) and downstream primer P4 (5′-AGTAGCAGTTTGCTTGGGTTGAG-3′). The targeted amplification product was assessed to be 100 base pairs in length. The total reaction volume employed was 20 *μ*L, comprising 10 *μ*L of 2 × SYBR Premix Ex Taq Ⅱ, 0.5 *μ*L of upstream and downstream primers (10 *μ*M), ddH_2_O 7 *μ*L. The reaction conditions were predenaturing 95°C 30 s, 95°C denaturing 5 s, and 60°C annealing extension for 30 s for 40 cycles. The melting curve is 95°C 5 s, 62°C 60 s, and 95°C continuous; the last 50°C 30 s ends the reaction. The temperature conversion rate was 20°C/s and the fluorescence signal was detected at the end of the extension of each cycle.

### 2.10. Clinical Sample Detection

Both RT-RAA methods, along with RT-PCR and RT-qPCR, were used to detect BCoV in 162 fecal samples, 50 nasal swab samples, and 30 tissue samples. The concordance rates of the results obtained from the various methods were calculated and compared. The consistency of the two RT-RAA methods was statistically analyzed by Cohen's “kappa” (*κ*) and *P*-value analysis.

## 3. Results

### 3.1. Optimization of Reaction Conditions for Real-Time RT-RAA Assays

**Figure 4:**
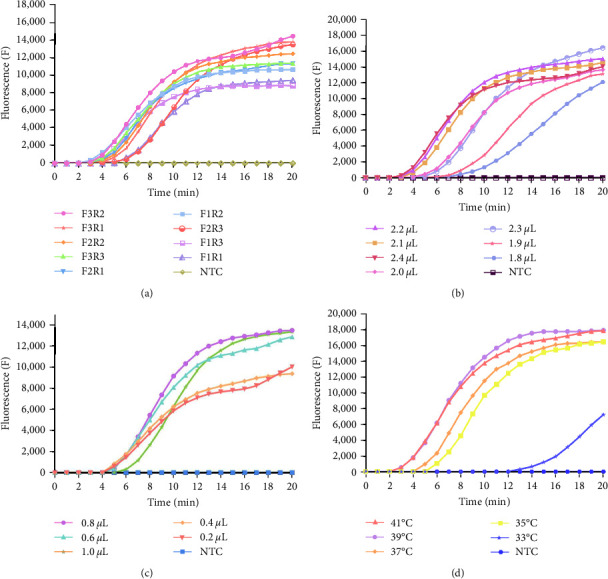
Optimization of reaction conditions for real-time RT-RAA experiments with BCoV. (a) Primer pairs screening, three pairs of upstream primers, and three pairs of downstream primers were designed to form primer pairs F1/R1, F1/R2, F1/R3, F2/R1, F2/R2, F2/R3, F3/R1, F3/R2, and F3/R3. DNAzyme-free water was used as a negative control. The primer screening revealed that the F2/R3 pair had the highest amplification efficiency. (b) Primer volume optimization: The primer volume was tested at five different gradients 1.6, 1.8, 2.0, 2.2, and 2.4 *μ*L with the optimal volume determined to be 2.2 *μ*L. (c) Probe volume optimization: The probe volume was also tested at five different gradients 0.2, 0.4, 0.6, 0.8, and 1.0 *μ*L with the optimal volume identified as 0.8 *μ*L. (d) Reaction temperature optimization: The reaction temperature was evaluated at five different gradients 33, 35, 37, 39, and 41°C with the optimal temperature determined to be 39°C. The results could be observed after a 20 min reaction period.

Nine pairs of primers (F1R1, F1R2, F1R3, F2R1, F2R2, F2R3, F3R1, F3R2, and F3R3) were screened, and their respective amplification curves are visualized in Figure 4(a). Nine primer pairs were tested for amplification, with the efficiency varying significantly among them. Notably, the F3R2 pair exhibited a fluorescence signal within 3 min and had the highest amplification efficiency. Consequently, the F3R2 primer pair was selected for further experimentation. As indicated in Figures 4(b) and 4(c), the strongest fluorescence signal and highest amplification efficiency were observed when the primer volume was set at 2.2 *μ*L and the probe volume at 0.8 *μ*L. These conditions constituted our optimized real-time RT-RAA reaction system, as summarized in Table 2. Temperature wise, when set within the range of 33–41°C, the amplification efficiency increased as the temperature rose. However, gains in efficiency became marginal upon reaching 39°C. Therefore, 39°C was chosen as the temperature for subsequent experiments, as shown in Figure 4(d).

### 3.2. Optimize Reaction Conditions for RT-RAA-LFD Assays

**Figure 5:**
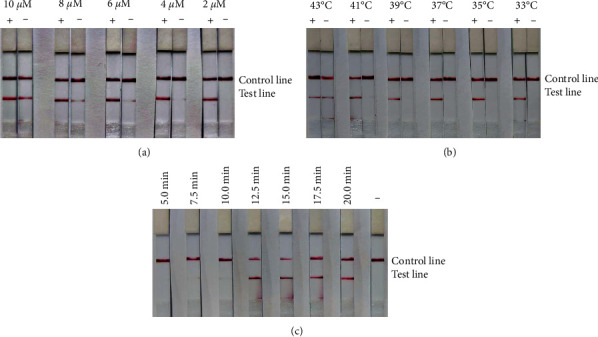
Optimizing reaction conditions for BCoV RT-RAA-LFD assay. (a) Optimization of primer and probe concentration. Primer concentrations were tested at five levels 2, 4, 6, 8, and 10 *μ*M as were probe concentrations. The optimal concentration for both primer and probe was found to be 2 *μ*M. RT-RAA-LFD experiments conducted with higher concentrations of primer and probe yielded false-positive results. (b) Optimization of reaction temperature. Temperatures were set at six gradients 33, 35, 37, 39, 41, and 43°C. The optimal temperature was determined to be 35°C, with observable results after 12.5 min of reaction time. When the temperature exceeded 39°C, the negative control group exhibited specific amplification. Additionally, as the temperature increased, the color intensity of the T line on the test strip for the negative control group became more pronounced, while that of the positive test strip gradually lightened. (c) Optimization of reaction time. Reaction times were tested at seven intervals 5, 7.5, 10, 12.5, 15, 17.5, and 20 min. Bands began to appear at 7.5 min and were clearest at 12.5 min.

In RT-RAA-LFD assay, the concentration of primer and probe was examined, as depicted in Figure 5(a). At concentrations of 2 and 4 *μ*M for both the primer and probe, the positive bands were clearly visible and no negative bands appeared. However, at concentrations ranging from 6 to 10 *μ*M, negative bands and false positives emerged, becoming more pronounced as the concentration increased.

Temperature optimization is illustrated in Figure 5(b). At temperature between 33 and 39°C, distinct positive bands were observed. In contrast, as temperatures increased from 39 to 43°C, the clarity of the positive bands diminished and nonspecific amplification occurred in the negative bands. Considering the high amplification efficiency and clarity of the positive bands specifically at 35°C, this temperature was selected for subsequent assays.

Reaction time optimization is presented in Figure 5(c). Bands began to appear at a reaction time of 7.5 min. Up to 12.5 min, the bands became clearer as the temperature increased. Beyond 12.5 min, the positive bands did not show significant changes. Therefore, the optimal reaction conditions were determined to be a temperature of 35°C for a duration of 12.5 min.

### 3.3. Real-Time RT-RAA Sensitivity, Specificity, and Reproducibility Assays

**Figure 6:**
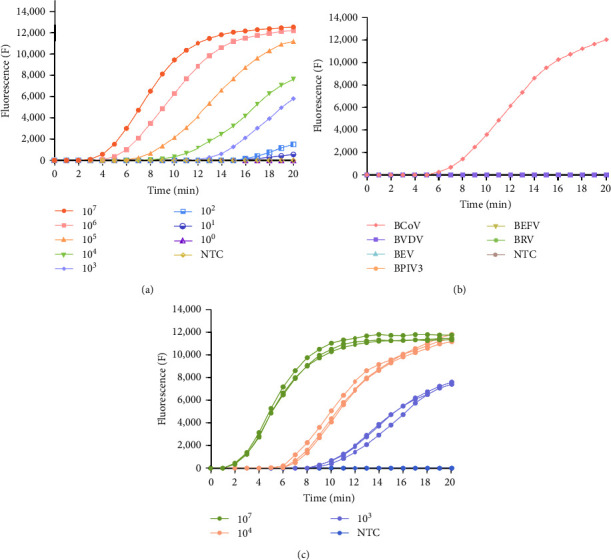
BCoV real-time RT-RAA sensitivity, specificity, and reproducibility assays. (a) Sensitivity assay. The prepared standard RNA was diluted to concentrations ranging from 1.46 × 10^7^ to 1.46 × 10^0^ copies/*μ*L. Water without DNAzyme served as the negative control. Real-time RT-RAA was able to detect as low as 1.46 × 10^1^ copies/*μ*L. (b) Specificity assay. Nucleic acids from BCoV, BVDV, BEV, BPIV3, BEFV, and BRV were used as templates. Water without DNAzyme was used as a negative control. Real-time RT-RAA demonstrated specific amplification only for BCoV. (c) Reproducibility Assay. Reproducibility experiments were conducted using three standard plasmid dilutions: 1.46 × 10^7^, 1.46 × 10^4^, and 1.46 × 10^3^ copies/*μ*L.

The sensitivity of real-time RT-RAA assay is depicted in Figure 6(a). The lowest detection level achieved was 1.46 × 10^1^ copies/*μ*L. The specificity test, as illustrated in Figure 6(b), revealed that only BCoV was specifically amplified by real-time RT-RAA; no fluorescence was observed for other viruses and negative controls. Importantly, the assay did not cross-react with BVDV, BEV, BPIV3, BEFV, and BRV. The reproducibility of the real-time RT-RAA assay is presented in Figure 6(c), demonstrating good assay reproducibility.

### 3.4. Results of RT-RAA-LFD Sensitivity, Specificity, and Reproducibility Assays

**Figure 7:**
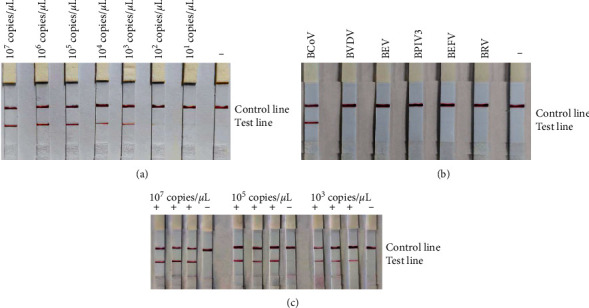
BCoV RT-RAA-LFD sensitivity, specificity, and reproducibility assays. (a) Sensitivity assay. A dilution series ranging from 1.46×10^7^–1.46 × 10^1^ copies/*μ*L was used, with water free of DNAzyme serving as a negative control. RT-RAA-LFD was able to detect as low as 1.46 × 10^2^ copies/*μ*L. (b) Specificity assay. RNA or DNA of BCoV, BVDV, BEV, BPIV3, BEFV, and BRV were used as templates. Water without DNAzyme served as a negative control. Only BCoV showed specific RT-RAA-LFD amplification. (c) Reproducibility assay. Reproducibility experiments were conducted using three standard RNA dilutions: 1.46 × 10^7^, 1.46 × 10^4^, and 1.46 × 10^3^ copies/*μ*L.

The sensitivity of the RT-RAA-LFD experiment is illustrated in Figure 7(a), where the lowest detectable concentration was 1.46 × 10^2^ copies/*μ*L. The specificity test is depicted in Figure 7(b); the results indicate that only BCoV was specifically amplified by RT-RAA-LFD. No amplification was observed for other viruses or the negative control. The method showed no cross-reactivity with BVDV, BEV, BPIV3, BEFV, and BRV. The reproducibility of the RT-RAA-LFD experiments is depicted in Figure 7(c), which demonstrates consistent and reliable results across trials.

### 3.5. RT-PCR and RT-qPCR Sensitivity Assays

**Figure 8:**
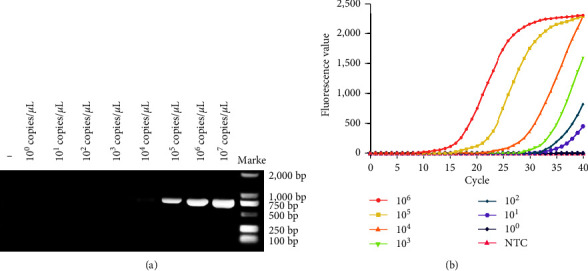
BCoV RT-PCR and RT-qPCR sensitivity assays. (a) RT-PCR sensitivity assay. A dilution series ranging from 1.46 × 10^7^–1.46 × 10^0^ copies/*μ*L was used, and water without DNAzyme served as the negative control. The RT-PCR assay was capable of detecting as low as 1.46 × 10^4^ copies/*μ*L. (b) RT-qPCR sensitivity assay. Dilutions ranged from 1.46 × 10^6^–1.46 × 10^0^ copies/*μ*L, and water without DNAzyme was used as a negative control. The RT-qPCR assay could detect as low as 1.46 × 10^1^ copies/*μ*L.

The sensitivity of the RT-PCR assay is illustrated in Figure 8(a) [32], where the lowest detection level was 1.46 × 10^4^ copies/*μ*L. Similarly, the sensitivity of the RT-qPCR assay is depicted in Figure 8(b) [33], with a lowest detection level of 1.46 × 10^1^ copies/*μ*L. The results from both the RT-PCR and RT-qPCR assays are consistent with previously reported data.

### 3.6. Clinical Sample Detection

In this study, 242 samples were tested using four different methods, as outlined in Table 3. The results of real-time RT-RAA and RT-RAA-LFD were consistent with those obtained via RT-PCR and RT-qPCR, as presented in Table 4. Real-time RT-RAA and RT-qPCR each identified 34 positive samples, yielding a positive detection rate of 14.05% (34/242). RT-RAA-LFD identified 31 positive samples, with a positive detection rate of 12.81% (31/242). In contrast, RT-PCR detected only 26 positive samples, resulting in a positive detection rate of 10.74% (26/242). The detection rates for real-time RT-RAA and RT-RAA-LFD were higher than that of RT-PCR but were similar to that of RT-qPCR. The kappa value of comparing real-time RT-RAA with RT-PCR and RT-qPCR are, respectively, 0.848 and 1, the kappa value of RT-RAA-LFD, comparing RT-PCR and RT-qPCR are 0.901 and 0.947, the kappa value >0.8, *P* < 0.001, both RT-RAA methods provided accurate detection results.

## 4. Discussion

Since its initial isolation from diarrheic calves in the United States in 1972, BCoV has been identified in several regions worldwide, including Europe, America, Oceania, Asia, and Africa. Studies have reported the disease in various countries including Italy, Sweden, the United Kingdom, Belgium, the Netherlands, Australia, Canada, Argentina, Korea, Japan, France, and China [11, 13, 34–37]. Genetic evolutionary analyses of reported BCoV and BCoV-like strains indicate the formation of three major evolutionary branches: the Americas–Asia subcluster, the European subcluster, and the vaccine subcluster (comprising prototype strains) [38]. These findings demonstrate the global distribution of BCoV. The economic implications of the disease are significant, impacting the treatment and prevention costs, loss of livestock, and the international trade of beef and dairy products, causing substantial economic losses to farmers worldwide [39–41]. In China, the Ministry of Agriculture and Rural Affairs (MARA) categorizes BCoV as a category three animal disease, referring to commonly occurring diseases that can cause significant economic losses and require control measures. Given the absence of a specific therapeutic drug for BCoV, the development of precise detection methods becomes critically important. This revision aims to maintain a formal and clear exposition of BCoV's history, impact, and the importance of detection in managing the disease.

Traditional BCoV detection methods are broadly classified into serological and molecular biology categories. Serological methods include enzyme-linked immunosorbent assays (ELISA), fluorescent antibody techniques (FA), virus neutralization tests, and immunocolloidal gold techniques [42, 43]. On the other hand, molecular biology approaches comprise techniques such as PCR, qPCR, multiplex PCR, and loop-mediated isothermal amplification (LAMP) [44–46]. Despite their prevalent use, these methods have limitations. ELISA, for example, does not require high temperatures but involves intricate processes and lengthy incubation periods, typically 3–4 hr, and is prone to variability and a significant number of false results. Molecular biology methods require costly equipment and involve thermal cycling, with procedures lasting 1–2 hr. LAMP, while efficient, demands precise primers and is susceptible to aerosol contamination that may result in false positives, alongside requiring relatively high temperatures for amplification, usually exceeding 60°C. Both serological and molecular biology methods typically demand sophisticated instrumentation and skilled personnel, considered to be time-consuming and labor-intensive.

The BCoV genome comprises five structural proteins: nucleocapsid protein (*N*), hemagglutinin-esterase protein (HE), spike protein (*S*), envelope protein (*E*), and membrane protein (*M*). Notably, the *N* protein plays a crucial role in viral replication, transcription, and translation. It is rich in antigens for virus-infected cells. Genetic analyses have indicated that the gene encoding the *N* protein is more considerably conserved compared to the other four structural genes, with *N* genes of different BCoV strains exhibiting over 96% similarity, compared to 93%–95% similarity for the other genes. Given these findings, the *N* gene is frequently utilized as a target in viral nucleic acid detection. In this study, we have developed rapid detection methods based on real-time RT-RAA and RT-RAA-LFD, targeting the conserved BCoV N gene.

In this study, we evaluated the sensitivities of two newly established RT-RAA assays. The real-time RT-RAA assay exhibited sensitivity up to 1.46 × 10^1^ copies/*μ*L, while the RT-RAA-LFD could detect up to 1.46 × 10^2^ copies/*μ*L. In comparison to RT-PCR and RT-qPCR assays, RT-RAA showed a 2–4 fold increase in sensitivity over RT-PCR and was comparable to RT-qPCR. RT-RAA-LFD was slightly less sensitive than RT-qPCR. We tested the specificity of both RT-RAA methods, against a variety of bovine viruses, including BVDV, BEV, BEFV, BPIV3, and BRV. In all cases, we found that only BCoV generated fluorescent signals, indicating its high specificity without cross-reactions. We further assessed the performance of these methods using 242 Bovine samples. Both real-time RT-RAA and RT-qPCR identified 34 positive samples, yielding a positive detection rate of 14.05%. RT-RAA-LFD identified 31 positive samples, for a rate of 12.81%, while RT-PCR detected 26, corresponding to a 10.74% positive rate. Notably, the specificity of real-time RT-RAA was 100% consistent with RT-qPCR results from clinical samples.

The real-time RT-RAA fluorescence instrument, weighing merely 1.5 kg, offers portability and a cost advantage over RT-PCR and RT-qPCR instruments. Conversely, the RT-RAA-LFD method does not need for specialized instrumentation; relying instead on a simple heating device, such as a water bath, with results that can be interpreted using test strips. Temperature profiling for both methods revealed that real-time RT-RAA achieved high amplification efficiency between 39 and 41°C, consistent with previous reports. RT-RAA-LFD, shows increasing amplification efficiency from 33 to 39°C, however, temperatures above 39°C lead to nonspecific amplification in the negative controls. We speculate that enzyme activity may be adversely affected at elevated temperatures. Both assays were functional at 33°C, although the reaction was slower and signal intensity weaker. Optimal conditions for real-time RT-RAA were found to be 39°C for 20 min, and for RT-RAA-LFD, 35°C for 17.5 min. Although RT-RAA-LFD was slightly less sensitive than real-time RT-RAA, its optimal reaction temperature was 4°C lower, and its reaction time was shorter. Given that RT-RAA-LFD does not require specialized equipment, it may even be possible to use body heat to initiate the reaction, making it an ideal choice for deployment in remote or under-resourced areas. However, the use of RNA as the reaction template in the RT-RAA method remains a limitation, as it restricts the broader application and field testing of the method. Presently, this technique is more appropriate for the grassroots laboratories in remote areas equipped with rudimentary instruments such as centrifuges and water baths. Nonetheless, the practical application of the RT-RAA method for on-site detection is a necessary development direction that requires further research and development, which to a certain extent, forms the foundation for future advancement.

RT-RAA effectively overcomes several limitations of RT-PCR and RT-qPCR methods. Employing lyophilization technology, the dry powder reagent offers enhanced stability and extended shelf-life, making it well-suited for transportation and preservation, and also avoids the loss caused by repeated freezing and thawing. Unlike RT-PCR and RT-qPCR, RT-RAA eliminates the need for a reverse transcription process, allowing RNA to directly participate in the reaction. Significantly, RT-RAA reduces the reaction time to less than 20 min, a substantial improvement over the 1–2 hr typically required by RT-PCR and real-time RT-PCR, thus offering a time efficiency of approximately 3–8 fold. Moreover, expensive instrumentation is not required for detection, in contrast to the equipment needed for RT-PCR and RT-qPCR. The results are also readily visualized. Real-time RT-RAA allows for fluorescence signal observation within 5 min, while RT-RAA-LFD enables direct result observation via a test strip. Both the real-time RT-RAA and RT-RAA-LFD assays ensure that the amplification product remains isolated with air until the end of the reaction. This eliminates the need for subsequent electrophoresis, purification, or other manipulations, thus minimizing the risk of cross-contamination. This is particularly important for achieving reliable visualization of the results. In terms of detection time, temperature requirements, specificity, and sensitivity, RT-RAA emerges as a novel rapid detection method.

## 5. Conclusions

In conclusion, the two RT-RAA assays developed in this study represent a significant advancement over traditional laboratory-based diagnostic methods. These innovative assays have the potential to enable the accurate detection of BCoV in resource-limited settings equipped, increasing accessibility to this vital diagnostic tool. They utilize compact, portable instruments, and feature straightforward operational procedures, thereby reducing the demand for highly specialized technical expertise among testing required personnel. Given their numerous advantages, RT-RAA methods are poised to set a new standard in rapid nucleic acid detection technology, potentially replacing traditional PCR methods. Their adaptability makes them particularly beneficial for use in remote or resource-limited settings. Moreover, it offers a reliable alternative for the detection and early diagnosis of BCoV, which is critical for effective monitoring, prevention, and control of BCoV disease.

## Figures and Tables

**Table 1:** The primers and probes, sequences of real-time RT-RAA primers and exo probe, RT-RAA-LFD primers and nfo probe.

Methods	Primers	Sequences (5′→3′)	Position of *N* (gene/bp)	
Real-time RT-RAA	Exo probe	ACCCAAGTAGCGATGAGGCTATTCCGAC[FAM-dT]A[THF]G[BHQ-dT]TTCCGCCTGGCACGG-C3Spacer	494–541	This study
	F1	TATGGCACCGATATTGACGGAGTCTTCTGG	412–441	This study
	R1	GAGCAGACCTTCCTGAGCCTTCAATATAGT	557–586	This study
	F2	GATATTGACGGAGTCTTCTGGGTCGCTAGT	421–450	This study
	R2	ATGCGCGTGAAGTAGATCTGGAATTAGGAG	584–612	This study
	F3	TCGCTAGTAACCAGGCTGATGTCAATACCC	442–472	This study
	R3	CAGAATTGGCTCTACTACGCGATCCTGCAC	629–658	This study

RT-RAA-LFD	Nfo probe	(FAM)ACCCAAGTAGCGATGAGGCTATTCCGACTA[THF]GTTTCCGCCTGGCACGG-C3Spacer	494–541	This study
	F	TCGCTAGTAACCAGGCTGATGTCAATACCC	442–472	This study
	R	(Biotin) GAGCAGACCTTCCTGAGCCTTCAATATAGT	557–586	This study

RT-PCR	P1	GAGCGTCCTTTGGAAATCGT	—	[32]
	P2	GCTTAGTTACTTGCTGTGGC	—	[32]

RT-qPCR	P3	TCGTTCTGGTAATGGCATCCT	—	[33]
	P4	AGTAGCAGTTTGCTTGGGTTGAG	—	[33]

FAM-dT: thymidine nucleotide carrying fluorescein; THF: tetrahydrofuran spacer; BHQ1-dT: thymidine nucleotide carrying black-hole Quencher-1; C3 spacer: polymerase extension blocking group; FAM: carboxyfluorescein.

**Table 2:** Real-time RT-RAA optimal reaction system and RT-RAA-LFD optimal reaction system.

Real-time RT-RAA		RT-RAA-LFD	
Reagent	Volume (*μ*L)	Reagent	Volume (*μ*L)
A buffer	25	A buffer	25
ddH_2_O	15.3	ddH_2_O	15.9
Upstream primer (10 *μ*M)	2.2	Upstream primer (2 *μ*M)	2.0
Downstream primer (10 *μ*M)	2.2	Downstream primer (2 *μ*M)	2.0
Exo probe (10 *μ*M)	0.8	Nfo probe (2 *μ*M)	0.6
Template	2	Template	2
B buffer	2.5	B buffer	2.5
Total	50	Total	50

**Table 3:** The results of real-time RT-RAA, RT-RAA-LFD, RT-PCR, and RT-qPCR assays in detecting suspected cases of BCoV infection in clinical samples.

Sample	Total	Real-time RT-RAA		RT-RAA-LFD		RT-PCR		RT-qPCR	
		Positive	Negative	Positive	Negative	Positive	Negative	Positive	Negative
Fece	162	17	145	16	146	14	148	17	145
Swab	50	11	39	10	40	9	41	11	39
Tissue	30	6	24	5	25	3	27	6	24
Total	242	34	208	31	211	26	216	34	208

**Table 4:** Coincidence rate of real-time RT-RAA, RT-RAA-LFD, RT-PCR, and RT-qPCR methods for BCoV detection.

	RT-PCR			RT-qPCR		
	Positive	Negative	Total	Positive	Negative	Total
Real-time RT-RAA	26	8	34 (14.05%)	34	0	34 (14.05%)
Positive	0	208	208	0	208	208
Negative	26 (10.74%)	216	242	34 (14.05%)	208	242
Total			Kappa = 0.848 *P* < 0.001			Kappa = 1 *P* < 0.001

RT-RAA-LFD	26	5	31 (12.81%)	31	0	31 (12.81%)
Positive	0	211	211	3	208	211
Negative	26 (10.74%)	216	242	34 (14.05%)	208	242
Total			Kappa = 0.901 *P* < 0.001			Kappa = 0.947 *P* < 0.001

## Data Availability

The dataset analyzed during the current study is available from the corresponding author upon reasonable request.

## References

[1] McNulty M. S., Bryson D. G., Allan G. M., Logan E. F. (1984). Coronavirus infection of the bovine respiratory tract. *Veterinary Microbiology*.

[2] Saif L. J., Jung K. (2020). Comparative pathogenesis of bovine and porcine respiratory coronaviruses in the animal host species and SARS-CoV-2 in humans. *Journal of Clinical Microbiology*.

[3] Siddell S. G., Anderson R., Cavanagh D. (2008). Coronaviridae. *Intervirology*.

[4] Boileau M. J., Kapil S. (2010). Bovine coronavirus associated syndromes. *Veterinary Clinics of North America: Food Animal Practice*.

[5] Cho K.-O., Halbur P. G., Bruna J. D. (2000). Detection and isolation of coronavirus from feces of three herds of feedlot cattle during outbreaks of winter dysentery-like disease. *Journal of the American Veterinary Medical Association*.

[6] Hodnik J. J., Ježek J., Starič J. (2020). Coronaviruses in cattle. *Tropical Animal Health and Production*.

[7] Cho Y.-I., Kim W.-I., Liu S., Kinyon J. M., Yoon K. J. (2010). Development of a panel of multiplex real-time polymerase chain reaction assays for simultaneous detection of major agents causing calf diarrhea in feces. *Journal of Veterinary Diagnostic Investigation*.

[8] Conrady B., Brunauer M., Roch F.-F. (2021). *Cryptosporidium* spp. infections in combination with other enteric pathogens in the global calf population. *Animals*.

[9] Kirchhoff J., Uhlenbruck S., Keil G. M., Schwegmann-Wessels C., Ganter M., Herrler G. (2014). Infection of differentiated airway epithelial cells from caprine lungs by viruses of the bovine respiratory disease complex. *Veterinary Microbiology*.

[10] Foster D. M., Smith G. W. (2009). Pathophysiology of diarrhea in calves. *Veterinary Clinics of North America: Food Animal Practice*.

[11] Bertoni E., Aduriz M., Bok M. (2020). First report of group A rotavirus and bovine coronavirus associated with neonatal calf diarrhea in the northwest of Argentina. *Tropical Animal Health and Production*.

[12] Beuttemmuller E. A., Alfieri A. F., Headley S. A., Alfieri A. A. (2017). Brazilian strain of bovine respiratory coronavirus is derived from dual enteric and respiratory tropism. *Genetics and Molecular Research*.

[13] Naciri M., Lefay M. P., Mancassola R., Poirier P., Chermette R. (1999). Role of *Cryptosporidium parvum* as a pathogen in neonatal diarrhoea complex in suckling and dairy calves in France. *Veterinary Parasitology*.

[14] Patarca R., Haseltine W. A. (2023). Intragenomic rearrangements involving 5′-untranslated region segments in SARS-CoV-2, other betacoronaviruses, and alphacoronaviruses. *Virology Journal*.

[15] Keha A., Xue L., Yan S., Yue H., Tang C. (2019). Prevalence of a novel bovine coronavirus strain with a recombinant hemagglutinin/esterase gene in dairy calves in China. *Transboundary and Emerging Diseases*.

[16] Vega V. B., Ruan Y., Liu J. (2004). Mutational dynamics of the SARS coronavirus in cell culture and human populations isolated in 2003. *BMC Infectious Diseases*.

[17] Alekseev K. P., Vlasova A. N., Jung K. (2008). Bovine-like coronaviruses isolated from four species of captive wild ruminants are homologous to bovine coronaviruses, based on complete genomic sequences. *Journal of Virology*.

[18] Amer H. M. (2018). Bovine-like coronaviruses in domestic and wild ruminants. *Animal Health Research Reviews*.

[19] Smith F. L., Heller M. C., Crossley B. M. (2022). Diarrhea outbreak associated with coronavirus infection in adult dairy goats. *Journal of Veterinary Internal Medicine*.

[20] Tsunemitsu H., el-Kanawati Z. R., Smith D. R., Reed H. H., Saif L. J. (1995). Isolation of coronaviruses antigenically indistinguishable from bovine coronavirus from wild ruminants with diarrhea. *Journal of Clinical Microbiology*.

[21] Zhang X. M., Herbst W., Kousoulas K. G., Storz J. (1994). Biological and genetic characterization of a hemagglutinating coronavirus isolated from a diarrhoeic child. *Journal of Medical Virology*.

[22] Majhdi F., Minocha H. C., Kapil S. (1997). Isolation and characterization of a coronavirus from elk calves with diarrhea. *Journal of Clinical Microbiology*.

[23] Hasoksuz M., Alekseev K., Vlasova A. (2007). Biologic, antigenic, and full-length genomic characterization of a bovine-like coronavirus isolated from a giraffe. *Journal of Virology*.

[24] Cui J., Li F., Shi Z.-L. (2019). Origin and evolution of pathogenic coronaviruses. *Nature Reviews Microbiology*.

[25] Su S., Wong G., Shi W. (2016). Epidemiology, genetic recombination, and pathogenesis of coronaviruses. *Trends in Microbiology*.

[26] Tao Y., Shi M., Chommanard C. (2017). Surveillance of bat coronaviruses in Kenya identifies relatives of human coronaviruses NL63 and 229E and their recombination history. *Journal of Virology*.

[27] Qin S., Xia X., Shi X., Ji X., Ma F., Chen L. (2021). Mechanistic insights into SARS-CoV-2 epidemic via revealing the features of SARS-CoV-2 coding proteins and host responses upon its infection. *Bioinformatics*.

[28] Vijgen L., Keyaerts E., Lemey P. (2006). Evolutionary history of the closely related group 2 coronaviruses: porcine hemagglutinating encephalomyelitis virus, bovine coronavirus, and human coronavirus OC43. *Journal of Virology*.

[29] Shen X.-X., Qiu F.-Z., Shen L.-P. (2019). A rapid and sensitive recombinase aided amplification assay to detect hepatitis B virus without DNA extraction. *BMC Infectious Diseases*.

[30] Fan X., Li L., Zhao Y. (2020). Clinical validation of two recombinase-based isothermal amplification assays (RPA/RAA) for the rapid detection of African swine fever virus. *Frontiers in Microbiology*.

[31] Wang Z.-H., Li P., Lin X. (2021). Application of portable real-time recombinase-aided amplification (rt-RAA) assay in the clinical diagnosis of ASFV and prospective DIVA diagnosis. *Applied Microbiology and Biotechnology*.

[32] Zhang W. Q., Zhang Y. C., Hu J. J. (2017). Whole genome sequence determination of bovine coronavirus BCV-Aks-01 Xinjiang southern Xinjiang strain and its molecular characterization. *Journal of Virology*.

[33] Shen F. Y., Yang J. L., Zhao G. M. (2016). Establishment and preliminary application of real-time fluorescence quantitative PCR detection method for bovine coronavirus SYBR greenI. *Chinese Journal of Veterinary Medicine*.

[34] Uhde F. L., Kaufmann T., Sager H. (2008). Prevalence of four enteropathogens in the faeces of young diarrhoeic dairy calves in Switzerland. *The Veterinary Record*.

[35] Shin J., Choe S. E., Park G.-N. (2022). Isolation and genetic characterization of a bovine coronavirus KBR-1 strain from calf feces in South Korea. *Viruses*.

[36] Symes S. J., Allen J. L., Mansell P. D. (2018). First detection of bovine noroviruses and detection of bovine coronavirus in Australian dairy cattle. *Australian Veterinary Journal*.

[37] Workman A. M., McDaneld T. G., Harhay G. P., Das S., Loy J. D., Hause B. M. (2022). Recent emergence of bovine coronavirus variants with mutations in the hemagglutinin-esterase receptor binding domain in U.S. cattle. *Viruses*.

[38] Kin N., Miszczak F., Diancourt L. (2016). Comparative molecular epidemiology of two closely related coronaviruses, bovine coronavirus (BCoV) and human coronavirus OC43 (HCoV-OC43), reveals a different evolutionary pattern. *Infection, Genetics and Evolution*.

[39] Burgstaller J., Obritzhauser W., Kuchling S., Kopacka I., Pinior B., Köfer J. (2015). The effect of bovine viral diarrhoea virus on fertility in dairy cows: two case-control studies in the province of Styria, Austria. *Berliner und Munchener Tierarztliche Wochenschrift*.

[40] Donovan G. A., Dohoo I. R., Montgomery D. M., Bennett F. L. (1998). Calf and disease factors affecting growth in female holstein calves in Florida, USA. *Preventive Veterinary Medicine*.

[41] Richter V., Lebl K., Baumgartner W., Obritzhauser W., Käsbohrer A., Pinior B. (2017). A systematic worldwide review of the direct monetary losses in cattle due to bovine viral diarrhoea virus infection. *The Veterinary Journal*.

[42] Socha W., Larska M., Rola J., Bednarek D. (2022). Occurrence of bovine coronavirus and other major respiratory viruses in cattle in Poland. *Journal of Veterinary Research*.

[43] Bertoni E. A., Bok M., Vega C., Martinez G. M., Cimino R., Parreño V. (2021). Influence of individual or group housing of newborn calves on rotavirus and coronavirus infection during the first 2 months of life. *Tropical Animal Health and Production*.

[44] Geng J., Niu Y., Wei L., Li Q., Gong Z., Wei S. (2020). Triplex qRT-PCR with specific probe for synchronously detecting bovine parvovirus, bovine coronavirus, bovine parainfluenza virus and its applications. *Polish Journal of Veterinary Sciences*.

[45] Tsunemitsu H., Smith D. R., Saif L. J. (1999). Experimental inoculation of adult dairy cows with bovine coronavirus and detection of coronavirus in feces by RT-PCR. *Archives of Virology*.

[46] Amoroso M. G., Lucifora G., Degli Uberti B. (2020). Fatal interstitial pneumonia associated with bovine coronavirus in cows from southern Italy. *Viruses*.

